# Supplementary material for: Complexome profiling of the Chlamydomonas psb28 mutant reveals TEF5 as an early PSII assembly factor
Source: Plant Cell. 2025 Mar 17;37(6):koaf055. doi: 10.1093/plcell/koaf055 (PMC12164590; doi:10.1093/plcell/koaf055)
Supplement: koaf055_Supplementary_Data [file koaf055_supplementary_data.zip › psb28_interactive.zip!/plots/intensity/qualityPlots/peptides/SDH3_Cre01.g020350.t1.2.html]

### Cre01.g020350.t1.2

Trivial name: SDH3  
SeqCoverage [median]: 30.50  
Mass: 21.66 kDa  
  
 

| Sequence | MissedCleavage | Mass [Da] | wtIntensity | muIntensity | Score |
| --- | --- | --- | --- | --- | --- |
| FPLGALSSITNR | 0 | 1274.70 | 11417.28 | 0.00 | 132.78 |
| HIVWDVSK | 0 | 982.52 | 388.75 | 610.47 | 75.85 |
| IGNQADR | 0 | 772.38 | 15409.01 | 24084.20 | 153.73 |
| KRPLSPDVMEIDNK | 2 | 1640.86 | 25294.46 | 35841.74 | 123.02 |
| NHLDLIK | 0 | 851.49 | 884.84 | 5995.13 | 116.88 |
| RPLSPDVMEIDNK | 1 | 1512.76 | 5545.57 | 9153.28 | 89.63 |
| TSLLELPK | 0 | 899.53 | 29841.90 | 61716.07 | 136.11 |
| TSLLELPKVEMSSK | 1 | 1560.84 | 788.60 | 1982.11 | 61.04 |
|  |  |  | 89570.4 | 139383.0 |  |
